# The Private Treatment of the Insane as Single Patients

**Published:** 1886-12

**Authors:** 


*The Private Treatment of the Insane as Single Patients.* By EDWARD EAST, M.R.C S., L.S.A., Member of the Medico - Psychological Association. London: J. & A. CHURCHILL. 1886.

The tide of public opinion having just now set in a direction opposed to public lunatic asylums, more attention will be devoted to the treatment of the insane in private houses. There can be no doubt that, for those cases of insanity in which there is a prospect of ultimate recovery, there are many reasons, both extrinsic and intrinsic to the patients themselves, why complete cure should be more probable and the subsequent contentment of the patients more certain if the treatment has been conducted throughout apart from a licensed institution; it being taken for granted that private asylums are, as for the most part they are indeed, models of perfection in management. For cases of incurable mental derangement, asylum treatment is no doubt in most instances the most convenient plan. Mr. East has published a book of plain and simple directions and suggestions for the treatment of the insane in private houses; he does not claim to have supplied a complete text-book; but the work is full of valuable practical information. To any practitioner who is acting or intends to act as medical attendant to a person of unsound mind, who is not under treatment in an asylum, we can most cordially commend the book.

